# Directed protein engineering identifies a human TIM-4 blocking antibody that enhances anti-tumor response to checkpoint inhibition in murine colon carcinoma

**DOI:** 10.1093/abt/tbae026

**Published:** 2024-09-23

**Authors:** Karla K Frietze, Kamala Anumukonda, Laura Padula, Natasha Strbo, Neil Goldstein

**Affiliations:** SkunkWorx Bio. 675 US-1 North Brunswick New Jersey, 08902, United States; SkunkWorx Bio. 675 US-1 North Brunswick New Jersey, 08902, United States; Department of Microbiology and Immunology, University of Miami Miller School of Medicine, 16000 NW 10th Ave Miami, FL 33136, United States; Department of Microbiology and Immunology, University of Miami Miller School of Medicine, 16000 NW 10th Ave Miami, FL 33136, United States; SkunkWorx Bio. 675 US-1 North Brunswick New Jersey, 08902, United States

**Keywords:** TIM-4, drug discovery, biologics, colorectal cancer, directed-protein engineering

## Abstract

**Background:**

T-cell immunoglobulin and mucin domain containing molecule-4 (TIM-4) is a scavenger receptor best known for its role in recognizing dying cells. TIM-4 orchestrates phagocytosis allowing for cellular clearance of apoptotic cells, termed efferocytosis. It was previously shown that TIM-4 directly interacts with AMPKα1, activating the autophagy pathway, leading to degradation of ingested tumors, and effectively reducing antigen presentation.

**Methods:**

This study sought to identify a novel human TIM-4 antibody that can prevent phagocytosis of tumor cells thereby allowing for more antigen presentation resulting in anti-tumor immunological response. Using phage display panning directed against human TIM-4, we engineered a novel human TIM-4 antibody (SKWX301). Combination of in vitro phagocytosis assays and cell viability assays were used to test functionality of SKWX301. To examine the effect of SKWX301 in mouse models, we employed a syngeneic mouse model. CT26 cells were subcutaneously injected into BALB/c mice and tumor growth and mouse survival were analyzed.

**Results:**

SKWX301 can prevent human macrophage phagocytosis of cancer cells in vitro. Combination of low dose SKWX301 and anti-PD1 antibody significantly inhibited tumor growth and increased overall survival in mice. This demonstrates that SKWX301 is effective in both human in vitro models and mouse in vivo models.

**Conclusion:**

Our study has demonstrated a rapid antibody discovery approach and identified a novel human TIM-4 antibody that can serve as a therapeutic for antitumor immunity to improve cancer therapy.

## Teaser

Statement of Significance: We developed a rapid antibody discovery program using directed-protein engineering to develop a first in class anti-human T-cell immunoglobulin and mucin domain containing molecule-4 (TIM-4) therapeutic antibody (SKWX301). SKWX301 binds human TIM-4 to block efferocytosis and effectively prevents tumor growth in a cancer murine syngeneic mouse model when combined with anti-PD-1.

## Introduction

Targeting macrophages for anti-tumor activity is an attractive approach for immunotherapy. There are distinct mechanisms for targeting macrophages for the utility of anticancer approaches; one approach involves targeting the “don’t eat me signal” (e.g. CD47) on live tumor cells to increase macrophage mediated phagocytosis of tumor cells and thereby promoting destruction of tumor cells. On the other hand, blocking “eat me signals” on apoptotic tumor cells can increase immune activation by allowing for efficient antigen presentation and therefore immune activation (reviewed in [1]). The differentiated approaches to harness macrophages for anti-tumor activity are promising strategies for immunotherapy. In this study we aim to develop a novel therapeutic against T-cell immunoglobulin and mucin domain containing molecule-4 (TIM-4) to block “eat me signals” on apoptotic cells. TIM-4 belongs to the T-cell immunoglobulin and mucin domain (TIM) gene family, comprising three members in human (TIM-1, TIM-3, and TIM-4) and eight members in mouse (TIM 1-8), known for their roles in immunoregulation (reviewed in [2–4]). TIM family members are cell surface type I membrane protein, containing an N-terminal cysteine rich IgV-like domain, a mucin domain, a transmembrane domain and an intracellular domain [5]. Unique to TIM-4 is that it contains an arginine-glycine-aspartic (RGD) motif in its extracellular domain, TIM-4’s RGD motif that has been shown to utilize integrins for phagocytosis [6]. TIM-4 mucin domain is longer than TIM-1 and TIM-3 and is rich in serines, threonines, proline residues [7]. Both TIM-1 and TIM-3 contain tyrosine residues in their cytoplasmic domain to allow for intracellular activation while TIM-4 lacks tyrosine residues and has been shown to be largely dispensable for efferocytosis [8]. The unique structure of TIM-4 differentiates it from TIM-1 and TIM-3. Several studies have linked the TIM family to development of allergic disease and autoimmunity [9–11]. TIM-4 was originally identified for its interaction with TIM-1 in regulation of T-cell proliferation [12]. TIM-4 expression has been shown to be restricted to antigen presenting cells (APCs), implicating its role in regulating immune functions [12]. TIM-1 and TIM-4 have both been shown to have critical roles in binding phosphatidylserine (PS) and mediating uptake of apoptotic cells [13, 14].

Removal of apoptotic cells is necessary to maintain immune homeostasis and prevent superfluous immune activation (reviewed in [15]). However, in cancer, the rapid removal of dying tumor cells can lead to immune tolerance due to decreased antigen presentation. In a study looking at patient samples of non-small cell-lung cancer (NSCLC), TIM-4 expression was found to be significantly higher in tumors compared to adjacent tissues [16], suggesting active efferocytosis. Furthermore, TIM-4 expression is significantly upregulated on professional APCs in the tumor microenvironment due to the increased release of danger-associated molecular patterns (DAMPs) [17]. Mechanistically, TIM-4 has been shown to directly interact with AMPKα1 to mediate degradation of dying tumor cells through autophagy. Tumor associated efferocytosis has been shown to contribute to reduced antigen presentation, which effectively impairs cytotoxicity T lymphocyte (CTL) responses thereby inhibiting the antitumor effects of chemotherapy [17]. Baghdadi et al., showed that blocking TIM-4 activity using a mouse TIM-4 antagonist antibody (RMT4-53) in combination with various chemotherapy agents and/or cancer vaccines improved the efficacy of anti-tumor therapies, leading to reduced tumor size and increased survival in mouse models [17, 18]. It has also been shown that even in the absence of a chemotherapeutic or cancer vaccine, TIM-4 blockade (anti-mouse TIM-4) combined with an antibody against programmed death-1 (PD-1) results in robust synergistic effects on tumor growth inhibition and overall survival in a syngeneic mouse model of colon cancer [19]. Together, this data highlights a novel therapeutic strategy to improve the efficacy of current immunotherapies by capitalizing on the role of TIM-4 immunoregulation.

The role of TIM-4 in efferocytosis is critical for normal immune modulation; however when patients undergo classical cancer therapies such as chemotherapy or radiation, the rapid clearance of dying tumor cells results in ineffective antigen presentation leading to a dampened immune response (reviewed in [20]). TIM-4 also modulates distinct effector arms (i.e., CD8+ cytotoxic T cells) of the adaptive immune response, implicating a further role in T cell regulation that may prove beneficial when targeted alongside T cell therapies, even in the absence of chemotherapy or radiation. Resistance to immunotherapy, such as checkpoint inhibitors like anti-PD-1 or PD-L1, is a clinical challenge that cuts across all of oncology and calls for new approaches that can increase immune activation to improve rates of response. Use of biologics for cancer therapy have the advantage of target specificity, selectivity, binding affinity all while reducing off target toxicity. In this study, we leverage our rapid antibody discovery platform to engineer a novel human anti-TIM-4 antibody that potently inhibits efferocytosis, thus improving tumor cell antigen presentation and immune cell infiltration. Finally, we show that blockade of TIM-4 with SKWX301 successfully potentiates treatment with anti-PD-1 antibody to inhibit tumor growth and improve survival *in vivo*.

## Methods

### Phage display

SKWX301 was identified by biopanning against human TIM-4 using a high diversity phage display library expressing fully human single domain antibodies and peptides of 20 amino acids in length. Phage display single domain antibody libraries consisted of a human variable heavy chain framework with each of the complementary-determining regions (CDRs) randomized. Briefly, human TIM-4 (Sino Biological) and hexahistidine (6x His) peptide were coated onto high-capacity microtiter plates overnight at 4 °C and blocked with nonfat milk in PBS at room temperature. Aliquots of the peptide or antibody library were added to 6x His coated wells and the plates were incubated at room temperature for 60 min (negative pan). Unbound phage were removed and added to the TIM-4 coated well and the plates incubated at room temperature for 120 min (positive pan). Following the pan, each well was washed thirteen times with PBS and the phage eluted with a glycine/HCl solution containing bovine serum albumin (pH 2.2) for 3–5 min. The eluted phage from each library were pooled, neutralized with 1 M Tris/HCl (pH 8.0) and added to a log-phase *Escherichia coli* TG1 in 2 YT-G (medium containing 2% glucose) for 60 min with constant shaking. Helper phage (M13K07; NEB) and carbenicillin were then added and the cells are incubated for an additional hour. The cells were pelleted and resuspended in 2 YT-CK (medium containing carbenicillin and kanamycin) and incubated overnight at 37 °C with constant shaking. The next day, the infected bacterial cells were centrifuged and the pellet discarded. The supernatant containing the phage was precipitated with one-fourth volume of 20% polyethylene glycol-8000 in 1.6 M NaCl by incubation on ice for 60 min. The precipitate was centrifuged and the phage pellet resuspended in 1 ml of PBS. The phage was then used for the next round of panning. After 3 rounds, the phage were stored at -80 °C.

### Phage ELISA

A phage ELISA was used to identify anti-TIM-4 positive peptides and antibodies. Briefly, eluants from round 3 were cloned on 2XYT plates with 100 μg/ml carbenicillin. Individual colonies were picked, added to 96-well cluster plates containing 2X YT medium and carbenicillin and grown overnight at 37 °C to make master stocks. 50 μl of master stock was transferred to another set of cluster plates containing 2 YT-G and helper phage and incubated with constant shaking for 3 h at 37 °C. The cultures are centrifuged, the supernatant discarded, and the bacterial pellet is resuspended in 2 YT-CK medium to be incubated overnight at 37 °C. The cells were removed by centrifugation, and the supernatants used in ELISA assays. Each well of a high capacity immunoplate was coated with TIM-4 or 6x His overnight at 4 C. The wells were blocked with NFM-PBS at room temperature for 60 min. Phage were then added and the plates incubated at room temperature for 2 h. After washing three times with PBS-Tween 20, plates were probed with an anti-M13 antibody conjugated to horseradish peroxidase (Sino Biological). After incubating for 60 min at room temperature, the plates were washed 5x in PBS-Tween 20, followed by an addition of TMB for 15–30 min at room temperature. The reaction was stopped by the addition of 1 N HCl and the OD measured using a microplate spectrophotometer at 450 nm (Varioskan, ThermoFisher). Positive binders (>2x over background) were picked and sequenced.

### Elisa

TIM-4 (human) (Sino Biological Inc:12161-H08H), TIM-4 (mouse) (Sino Biological Inc:12161-H08H), and TIM-3 (Sino Biological Inc: 50388-M08H) recombinant peptides were coated on a high capacity immunoplate overnight at 4 °C overnight in BupH Carbonate–Bicarbonate Buffer (Thermo Scientific: 28382). Plates were washed 3x in PBS-Tween 20 and blocked in 5% BSA in PBS-Tween 20 for 1 h at room temperature. Plates were washed 3x in PBS-Tween 20 and following antibody treatments were added: SKWX.

301, human IgG isotype control (Bio X Cell: BE0297), RMT4-53 (Bio X Cell: BE0171), and rat IgG isotype control (Bio X Cell: BE0090). Plates washed 5x in PBS-Tween 20, followed by incubation with secondary anti-human HRP antibody (Invitrogen: A24494) or anti-RAT HRP antibody (Jackson Immuno Research Labs: 112035003). Plates were wash 5x in PBS-Tween 20 followed by the addition of TMB ELISA Substrate Solutions (Thermo Scientific: 34024). The reaction was stopped by the addition of 1 N HCl and the OD measured using a microplate spectrophotometer at 450 nm (Glo-Max by Promega).

### Peptide synthesis

Peptide synthesis was completed by Lifetein. Purity of all peptides was above 90%. Peptides were reconstituted in DMSO at 10 mgs/ml.

### Phagocytosis assay with beads

For THP1 phagocytosis assay, 200,000 cells were treated with 50 nM PMA for 48 h, media was removed and replaced with RPMI containing 1% FBS. For phagocytosis assay in HEK293 stably expressing TIM-4, 200 000 cells were seeded on a 96 well plate overnight. Media was removed and replaced with DMEM media containing 1% FBS. 100 μg/ml of peptide was added to wells and incubated for 4 h. PS beads (Echelon Biosciences (P-B1PS-2)), FluoSpheres Carboxylate-Modified Microspheres (ThermoFisher Scientific (F8823), or Green Zymosan (FITC) (Abcam), were added to cells and incubated overnight. Cells were washed 5 times with PBS and read on a plate reader (Glo-Max by Promega) or visualized by microscopy using an inverted fluorescent microscope (Evos by ThermoFisher Scientific).

### Co-culture phagocytosis assay

On day one, A549 cells were seeded on a 24 well glass bottom plate. On day two, A549 cells were labeled using Qtracker 655 Cell Labeling Kit (ThermoFisher Scientific (Q25021MP). A549 cells were treated with 40 μM cisplatin (MedChemExpress (HY-17394)) overnight. THP1 cells were seeded and treated with 50 nM phorbol 12-myristate 13-acetate (PMA) on a 24 well glass bottom plate. On day three, THP1 media was replaced. Day four, THP1 media was removed and replaced with serum free RPMI. Drug treatments SKWX301 (5 μgs/ml) and cytochalasin D (20 μM) were added to THP1 cells for 4 h. Supernatant of A549 cells contained dead floating cells was removed and added to THP1 cells and incubated overnight. On day five, THP1 cells were washed three times to remove A549 cells that had not phagocytosed. Cells were labeled for 15 min in Hoechst stain followed by imaging using inverted fluorescent microscope (Evos by ThermoFisher Scientific) and quantified using a plate reader (Glo-Max by Promega).

### Cell lines

THP1 and CT-26, were obtained from ATCC and cultured according to ATCC guidelines. The HEK293-FT-Tim-4 was created using lentiviral TIM-4 particles from BPS Biosciences. Briefly 100,000 cells were plated in a 6 well plate. Lentiviral particles were added at a multiplicity of infection (MOI) equal to 20. Polybrene was used 8 μg/ml to aid in transfection efficiency. Cells were infected for 24 h followed by a complete media change. Recovery was allowed for an additional 24 h before puromycin selection took place. Puromycin selection was achieved at 2 μg/ml. Tim-4 expression was determined by RT-PCR followed by band extraction and sequencing, primers used to amplify TIM-4 and loading control GAPDH are as follows: Tim-4 forward: 5’AGAGACTGTTGTGACGGAGG, reverse: 5’GTCTTCCCTTCCATGCTGCA, GAPDH forward: 5’GTCTCCTCTGACTTCAACAGCG, reverse: 5’ACCACCCTGTTGCTGTAGCCAA.

### Predictive Modeling and docking

Antibody predictive modeling was performed with “Cyrus Bench: Protein Design and Structure Prediction, Powered by Rosetta”. Cyrus bench utilizes homology-based modeling to build structures followed by full atom Talaris2013 energy function to score protein conformations. The predictive software was derived from the Baker Lab at UW institute for Protein Design. SKWX301 modeling was performed using single chain homology modeling in Cyrus Bench computer aided design platform. Antibody image rendering was done using Maestro software by Schrödinger. PIPER docking was performed using Maestro software by Schrödinger. TIM-4 PBD file 5F7H chain A was used to dock to SKWX301. Thirty poses were analyzed with the most consistent pose chosen for illustration.

### Antibody construction, expression, and purification

SKWX301 plasmid construction was completed at GeneArt (ThermoFisher Scientific). SKWX301 was placed into the pcDNA3_4_Tar vector. Expression and purification were conducted by ThermoFisher Scientific using the ExpiCho expression system. Purification was done using MabSelect SuRe. Binding buffer: 25 mM Tris, 25 mM NaCl, pH 7.4), elution buffer: 100 mM Essigsaure, 100 mM NaCl, pH 3, collection tubes contain 10% 1.5 M Tris–HCl pH 7.4. Peptide concentration was measured (absorption at 280 nm) using a NanoDrop device. Purity was determined by Coomassie blue staining and immunoblot.

### Antibody half-life in C57BL/6 mice and ELISA

Charles River Laboratories performed antibody-half life study according to ARRIVE guidelines with the highest ethical practices. Three C57BL/6 mice were used per sampling group (8 groups total). Mice age was between 6-10 weeks. Body weight was recorded and behavior was monitored closely for any adverse effects to treatments. SKWX301 was provided in PBS at 24 h, all animals were euthanized following SOP#687. Dosing volume was 5 ml/kg (0.1 ml/20 g mouse), adjusted volume per body weight. Following dosing, sampling was done by collecting full volume blood by terminal cardiac puncture under isoflurane anesthesia for group 1 (no drug), for groups 2–7 .06 ml of blood by submental bleed under no anesthesia was collected and processed. Blood was processed serum and snap frozen. 25 μl of serum per sampling was use for ELISA. .6 μg/well of human TIM-4 antigen was coated on immunoplate. ELISA was performed as previously.

### In vivo tumor model

Male and female BALB/c mice (7–9 weeks old) were subcutaneously injected with 1x10^6^ CT-26 cells on day 0. All mice developed measurable tumors (median tumor size <200mm^3^) by day 7. Treatments were initiated on day 7 as follows: SKWX301 antibody (low = 25 μg; high = 250 μg) alone or in combination with anti-PD1 antibody (200 μg) (Cd279, Invivomab anti-mouse Pd-1 Clone: Rmp1-14). Anti-TIM-4 antibody, clone RMT4-53 (103253-090, Bio X Cell), was used as a reference control, as well as an anti-isotype human monoclonal antibody control (103479-666, Bio X Cell). All treatments were administered 3 times. Tumor size (mm^3^) was measured twice weekly, and it was calculated by multiplying the tumor length by the square of the tumor width divided by 2. All mice used in this study adhered to the standards of regulatory compliance and ethical guidelines, in line with the established protocols as directed by the Institutional Animal Care and Use Committee (IACUC). The study is reported according to ARRIVE guidelines.

### Flow cytometry

Cells were thawed on ice and resuspended in PBS + 1% BSA, aliquoted into plates (spleens 2 × 10^6^/well, all tumor cells/well) and washed 2x in PBS + 1% BSA. Cells were then resuspended and stained for viability and incubated on ice for 15 min. Cells were washed 2x in PBS + 1% BSA and incubated in FcBlock for 15 min on ice. Followed by staining with 50 μl of antibody stain mix for 30 min. Cells were then washed 1x with FACS buffer and resuspended in Perm/Fix and run on CYTOFLEX S. Live cells were gated for CD4 or CD8 for T cells and CD11b for monocyte/macrophages. Ki-67+ was used to assess proliferating cells, and CD44/CD62L analysis was used to stratify T cell subsets into naive, activated, and memory T cell populations; CD8 + CD107a + populations were quantified to assess degranulating CD8+ T cells (Antigen/Tumor-specific CTL). CD11b expressing cells were further classified based on expression of Ly6c. Ly6G and F4/8were used to distinguish monocytes, granulocytes, and macrophages. For antibody descriptions see supplementary table 1.

## Results

### Identification and screening anti-TIM-4 biologics for inhibition of phagocytosis

**Figure 1:**
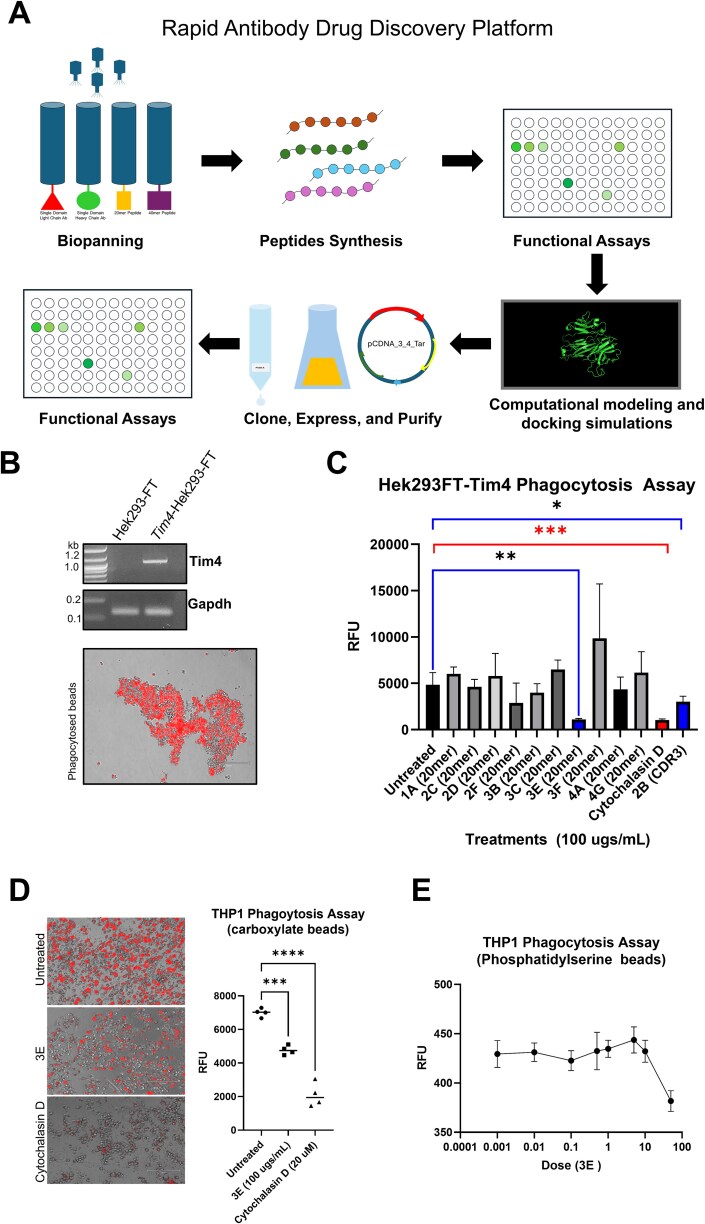
**Discovery platform and screening TIM-4 binders for inhibition of phagocytosis in human cell lines. A**) Workflow discovery platform. HEK293-FT cells were infected with lentiviral TIM-4 particles. Stable TIM-4 expression was confirmed by **B**) RT-PCR using primers against Tim-4 mRNA and Gapdh mRNA. Full length image of agarose gel available in supplementary fig. 1. Below: Representative image of *Tim-4-*HEK293-FT cells ability to phagocytose using carboxylate beads. **C**) Scale bar represents 300 μm. C) for THP-1 phagocytosis assay, cells were plated on a 96 well plate and treated with 100 μg of peptide or 20 μM of cytochalasin D. Fluorescence from phagocytosed beads was measured using a plate reader. Statistics were done using a one-way ANOVA followed by a multiple comparisons test comparing each sample to “untreated” control (*P >* .05 = ^*^, *P* > .01 = ^**^, *P >* .001 = ^***^). Blue bars represent peptides that were successful at blocking phagocytosis, red bar used for cytochalasin D control. **D**) THP1 phagocytosis assay, done the same as above. **E**) Dose curve for THP1 cells treated with fluorescent PS beads.

To identify biologics that bind to TIM-4 we utilized our rapid antibody discovery platform (Fig. 1A) Phage display was used to identify novel anti-TIM-4 biologics that could block phagocytosis. Phage display libraries containing peptides with lengths of twenty amino acids (20mer), as well as human single domain antibody libraries with fully randomized CDRs were used. Phage display panning identified both antibodies and 20mer peptides that bound human TIM-4 recombinant peptide.

CDRs are hypervariable domains in immunoglobulin (Ig) that bind to specific antigens. On an antigen receptor there are three CDRs (CDR1, CDR2, and CDR3) on the variable domain [21, 22]. The CDR3 loop has been shown to have a primary role in antigen–antibody interactions (reviewed in [23]). To test the anti-TIM-4 antibodies and peptides pulled out of phage display biopanning ability to inhibit phagocytosis, the CDR3 region of the candidate antibodies and the 20mer peptides were synthesized and tested using an *in vitro* phagocytosis assay. Synthetic peptides representing the CDRs of antibodies have previously been shown to be bioactive, allowing for more efficient production and a cost-effective method for screening [24]. For this assay, we developed a HEK293-FT cell line that overexpresses TIM-4 (Fig. 1B, Supplementary Fig. 1). 293 T cells overexpressing TIM-4 have previously been shown to successfully phagocytose apoptotic thymocytes, recapitulating TIM-4’s role in macrophages while allowing for the specific study of TIM-4 without interference of other TIM family members [25], specifically TIM-1 which has also been shown to bind PS [13, 14]. Therefore, to rapidly and specifically screen anti-TIM-4 peptides and CDR3 derived peptides, HEK293-FT overexpressing TIM-4 cells were used to determine the peptides anti-phagocytic activity. Although TIM-4 is best known for its role in binding PS on apoptotic cells to induce efferocytosis, TIM-4 is more broadly defined as a scavenger receptor due to its additional functions in phagocytosis of exogenous particles (e.g., bioparticles form *E.coli*, *S.aureus*, zymosan A, styrene and crystals from carbon nanotubes) [26, 27]. Therefore, for initial screening purposes we used carboxylate beads, zymosan particles, and PS beads to measure phagocytic engulfment. Cytochalasin D, a potent inhibitor of actin polymerization and therefore phagocytosis, was used as a reference control. The CDR3 peptide of candidate “2B” and the anti-TIM-4 peptide “3E” both successfully inhibited phagocytosis of fluorescently labeled beads (Fig. 1C). To validate whether peptide 3E also inhibited phagocytosis in macrophages, a THP1 phagocytosis assay was used. Peptide 3E also successfully inhibits phagocytosis in THP1 cells (Fig. 1D). Notably, peptide 3E exhibits stronger activity in HEK293-FT-Tim-4 cells than in THP1 cells, suggesting that other TIM family members and/or proteins may be involved in macrophage phagocytic uptake. To further validate that peptide 3E could not only inhibit phagocytosis of carboxylate beads but could inhibit beads mimicking apoptotic cells, fluorescently labeled PS beads were used. Peptide 3E was effective in limiting phagocytosis of PS coated beads in THP1 cells (Fig. 1E).

### Directed protein engineering of a human anti-TIM-4 antibody

**Figure 2:**
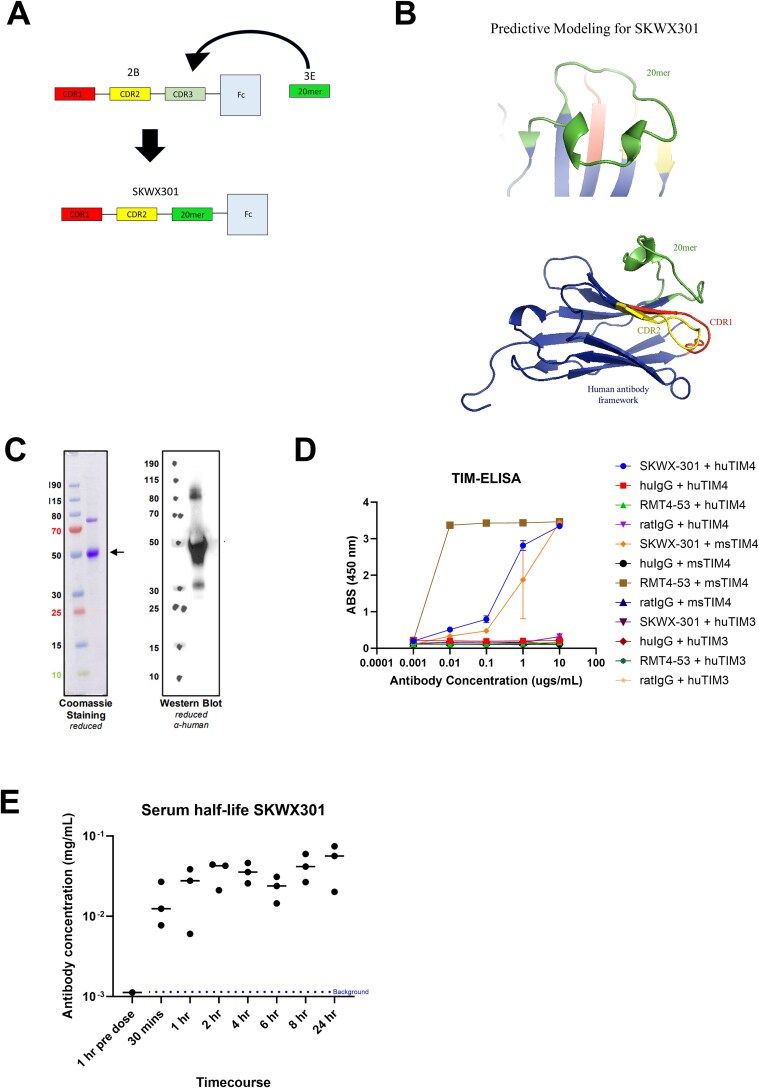
**Directed protein engineering of a human anti-TIM-4 antibody and binding confirmation. A**) Cartoon diagram of 20mer peptide being placed in CDR3 of TIM-4 antibody identified by phage display. **B**) Predictive modeling of 20mer peptide in CDR3 region using Cyrus bench single chain de novo modeling software. **C**) Coomassie SDS-PAGE (left) depicting SKWX301 purified protein, western blot (right) depicting purified SKWX301 probed with anti-human antibody. Image was provided by ThermoFisher scientific during their purification process. D) ELISA, recombinant TIM peptides were immobilized, and antibody treatments were added. SKWX301 specifically binds directly to human and mouse TIM-4 and not TIM-3. E) Serum half-life was determined from C57BL/6 mice that were injected with SKWX301, blood was collected and processed for serum according to time course shown in graph. ELISA was performed as previously with human TIM-4 recombinant protein used as an antigen for detection.

Although the 20mer peptide denoted as “3E” was identified as the lead candidate for TIM-4 inhibition, peptides are highly susceptible to degradation and rapid clearance, presenting significant stability concerns as a therapeutic agent [23, 24]. Thus, to maximize biological activity of an anti-TIM-4 biologic while preserving stability characteristics of a full antibody, we took an anti-TIM-4 antibody identified via phage display (2B) and replaced its CDR3 with the peptide sequence of 3E (Fig. 2A). Therefore, we had an antibody with CDR1 and CDR2 regions that were human TIM-4 specific with a highly potent 3E peptide in place of CDR3 to maximize its performance, creating a single domain TIM-4 antibody with a human IgG1 Fc. The resulting antibody was referred to as SKWX301. Using Rosetta protein design and modeling software provided by Cyrus Biotechnology, we performed predictive modeling of SKWX301 (Fig. 2B). Interestingly, where peptide 3E was inserted, there is noticeable secondary structure containing two alpha helical structures. By directed protein engineering, we have created a novel secondary structure within the critical antibody binding CDR3 loop. SKWX301 purified antibody is shown by Coomassie and western blot (Fig. 2C). A band corresponding can be detected at the predicted weight of 43 kDA (comprising single domain antibody 15 kDA plus Fc (26 kDA)), however of note SKW301 also shows a detectable band around 80 kDA even under reducing conditions, suggesting possible antibody dimerization and/or aggregation (Fig. 2C).

To confirm that SKWX301 binds TIM-4, we performed an ELISA using human and mouse TIM-4 recombinant peptides. Our negative controls consisted of human TIM-3 and isotype controls; as a positive control, we used RMT4-53, which is an antibody directed against mouse Tim-4 [13]. SKWX301 specifically binds to both human and mouse TIM-4, but not TIM-3 or isotype controls, confirming that specificity for TIM-4 was retained. Interestingly, we observed that RMT4-53 binds tightly to mouse TIM-4, but no binding to human TIM-4 was observed (Fig. 2D). To characterize antibody stability in a mouse model we used C57BL/6 mice and injected SKWX301. ELISA performed on serum collected from mice samples show that the antibody can still be detected at least 24 h after dosing (Fig. 2E).

### SKWX301 reduces efferocytosis in THP1 cells

**Figure 3:**
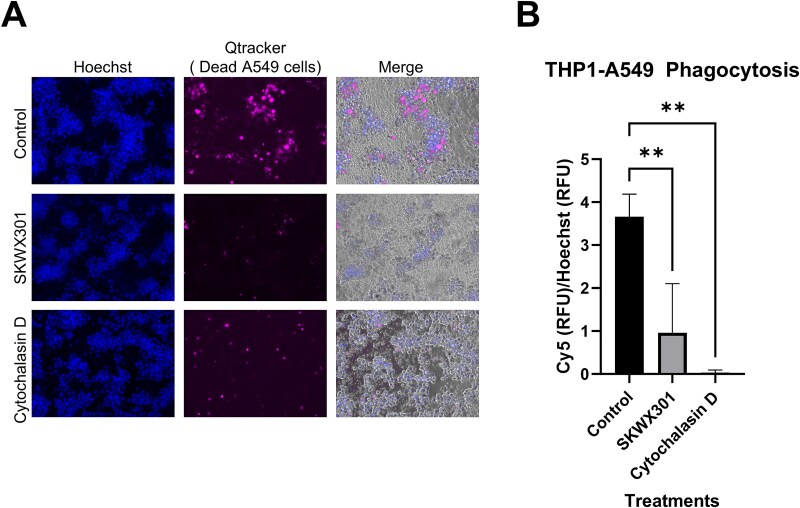
**SKWX301 reduced efferocytosis in THP1 cells.** A549 cells were labeled with Qtracker and treated with 40 μM of cisplatin overnight. Dead A549 cells were placed with PMA treated THP1 cells and efferocytosis was monitored. THP1 cells were washed three times and cells were visualized by microscopy **a)** and quantified using a plate reader, **B**). Cells that had been phagocytosed by THP1 were indicated by fluorescent signal at 655 nM. Normalization of phagocytosed A549 cells was normalized to Hoechst staining. Cytochalasin D limits phagocytosis 100% therefore fluorescence obtained from wells treated with cytochalasin D was used as a baseline for fluorescence and subtracted from all wells. Statistics were done using a one-way ANOVA followed by a multiple comparisons test comparing each sample to “untreated” control (*P >* .05 = ^*^, *P >* .01 = ^**^, *P >* .001 = ^***^).

To determine if SKWX301 can circumvent the “eat me” signal by blocking TIM-4 receptor binding to apoptotic cancer cells, we established a novel phagocytosis assay. Briefly, lung epithelial adenocarcinoma cells (A549 cells) were fluorescently labeled and subjected to a high concentration of cisplatin to mimic chemotherapy-induced cytotoxicity. Dead fluorescently labeled A549 cells were collected and incubated with THP1 macrophages to phagocytose dead A549 cells. THP1 macrophages were washed, imaged, and fluorescence was quantified on a plate reader. When dead A549 cells were incubated with THP1 macrophages in the presence of SKWX301, phagocytosis was significantly inhibited, as measured by 3.8 fold reduction in THP1 fluorescence compared to untreated control cells (Fig. 3).

### Low dose SKWX301 enhances the efficacy of anti-PD1 antibody treatment in a mouse model of colon carcinoma

**Figure 4:**
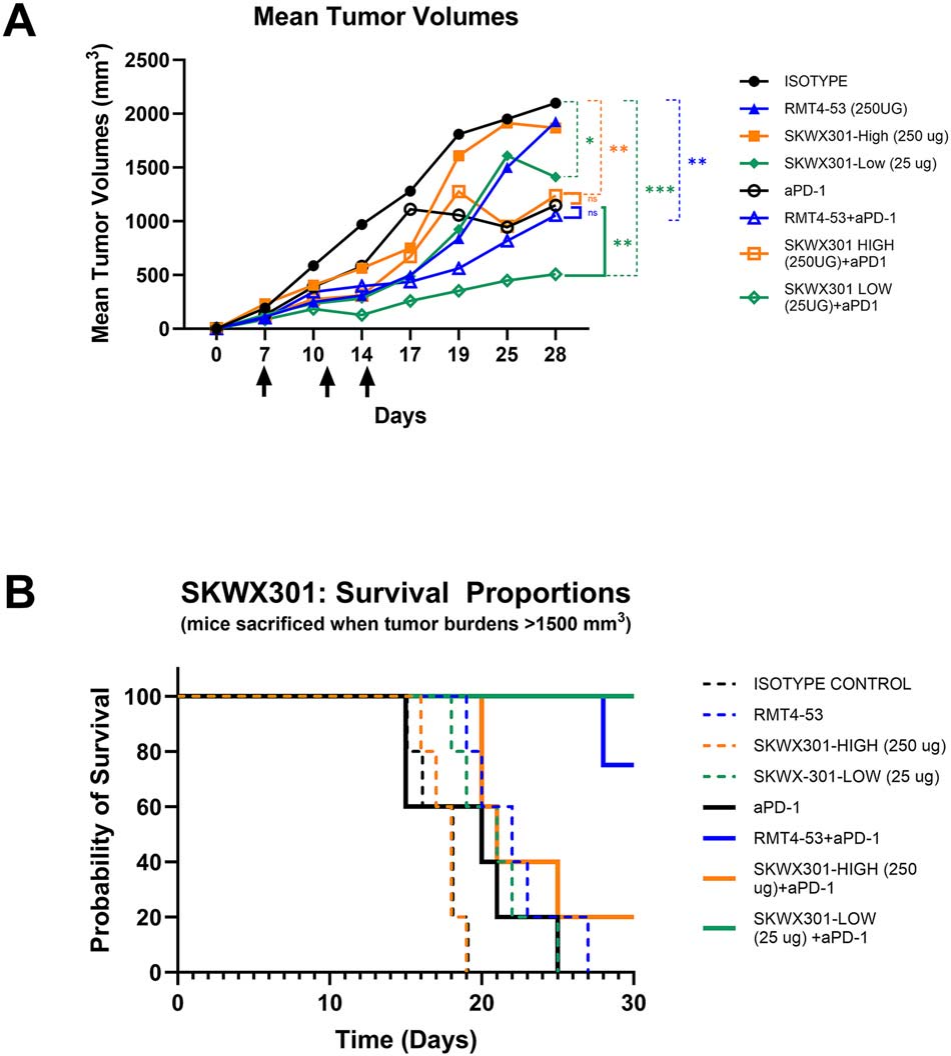
**Combination of SKWX301-low and anti-PD-1 antibody significantly inhibits tumor growth and improves survival. A) Tumor volume** measurements were evaluated twice weekly. The dosing schedule is indicated by black arrows. Statistical analysis is represented as dotted lines in treatments compared to isotype control, solid lines represent comparisons done to anti-PD-1 treatment, N = 5/treatment group; ^*^  *P >* 0.05, ^**^  *P >* 0.01, ^***^  *P >* 0.001. **B) Survival analysis**, dotted lines indicate single agent treatment, solid lines indicate treatment given in combination with anti-PD-1.

To investigate whether SKWX301 exhibits anti-cancer activity *in vivo*, we utilized a syngeneic mouse model of colon carcinoma. Previously, Baghdadi et al, showed that blockade of TIM-4 in combination with chemotherapeutics enhanced tumor-specific cytotoxic T lymphocyte responses [12]. Additionally, antibody blockade of Tim-4 improves anti-PD-1 efficacy in murine models of peritoneal carcinomatosis using colon carcinoma lines [14]. In colorectal cancer (CRC), the majority of patients, especially those with microsatellite-stable tumors, do not show clinical response to checkpoint inhibition alone [28, 29]. Thus, there is a great need for new therapeutic approaches that can restore the CRC tumor microenvironment to one that is responsive to immune checkpoint blockade. Thus, we sought to determine whether SKWX301 could sensitize a colon carcinoma to an anti-PD-1 inhibitor. To test this, BALB/c mice were subcutaneously injected with 1x10^6^ CT-26 cells on day 0. All mice had developed measurable tumors (median tumor size 200 mm3) by day 7, when they initiated treatment with SKWX301 at a low (25 μg) or high (250 μg) dose in combination with anti-PD1 antibody (200 μg). Anti-mouse Tim-4 (clone RMT4-53) was used as a reference control. SKWX301 (low dose) or anti-PD1 antibody monotherapy each exert a modest impact on tumor growth; however, dramatic reductions in tumor volume were observed when low dose SKWX301 and PD1 antibody were combined (2.3-fold reduction in tumor volume compared to anti-PD-1 treatment, and a 4-fold reduction compared to isotype controls) (Fig. 4A). This demonstrates that SKWX301 synergizes with checkpoint inhibition *in vivo* to inhibit tumor growth. Further, combination treatment with anti-TIM-4 and anti-PD-1 antibodies improved survival compared to anti-PD-1 alone, with all mice treated with anti-PD-1 monotherapy succumbing to disease by day 25 (Fig. 4B). In fact, 100% of SKWX301-low plus anti-PD-1 treated animals survived until endpoint (Fig. 4B). Together this data shows that TIM-4 blockade improves efficacy of anti-PD-1 to reduce tumor volumes and improve survival from murine colon carcinoma. Worth mentioning, SKWX301 was designed to harbor specificity to human TIM-4 but clearly exhibits biology activity in mouse models of disease.

To investigate how TIM-4 inhibition affects myeloid and T-cell populations, we performed flow cytometry analyses on spleens from the mice treated with the various antibody combinations. Previously, it has been shown that TIM-4 blockade in mouse xenografts did not significantly affect spleen lymphocytes and had a limited effect on tumor lymphocyte CD8 + T cells [13]. Consistent with this, we found no significant changes to myeloid cell, CD8+ T-cell, or CD4+ T-cell subsets in animals treated with SKX301 (Supplementary Fig. 2).

## Discussion

In this study, we sought to engineer a novel human anti-TIM-4 antibody with potent biological activity to act as a potentiator of checkpoint inhibition in cancers the exhibit innate or adaptive resistance to immunotherapy. Using phage display biopanning to identify biologic binders of human recombinant TIM-4, we identified a peptide that potently inhibits phagocytosis as well as a single domain human antibody with specificity for TIM-4 but reduced phagocytic activity. To harness both of these attributes in the same molecule, we employed a directed protein engineering strategy to combine the TIM-4 specific antibody backbone with the 20mer peptide, which we inserted into the CDR3 region. The resulting antibody, SKWX301, binds specifically to both human and mouse TIM-4 and inhibits efferocytosis in human cells (Fig. 2C, Fig. 3). Additionally, we have rapidly identified, developed and engineered a single domain antibody. Single domain antibodies have several advantages, beginning with their small size of roughly 15 kDA. The small size allows for it to be easily cloned and recombinantly expressed as a monomeric antibody that maintains antigen binding (reviewed in [30]). This allows for ease in modifications, production, and purification. Single domain antibodies small size also has been shown to have better tumor penetration [31, 32]. However, despite the advantages of a single domain antibody one drawback is a short half-life in serum due to rapid renal clearance [33], traditional single domain antibodies have been shown to have a half-life of around .5 to 2 h [34]. However, our data presented here show that we can still detect SKWX301 after 24 h post injection (Fig. 2E), this is likely due to adding an Fc fragment increasing its size roughly 3-fold, thus increasing stability, making it an attractive candidate for a therapeutic. Finally, our approach demonstrates the utility of synthesized peptides for rapid screening, providing an accelerated approach for antibody drug discovery, and directed engineering to create single domain antibodies for therapeutic use.

Antagonist antibodies of immune checkpoint inhibitor PD-1 have been shown to be an effective anti-cancer treatment in a wide variety of cancer types (reviewed in [35]). We sought to enhance the immunological potential of anti-PD-1 treatment by combining it with our anti-TIM-4 antibody. We show that combination treatment with anti-PD-1 and SKWX301 (low dose, 25 μg) works synergistically to drastically reduce tumor progression in a murine model of colon carcinoma, a disease characterized by high levels of resistance to checkpoint inhibition. Furthermore, when comparing RMT4-53 with SKWX301, combination treatment with RMT4-53 and anti-PD-1 was comparable to treatment with anti-PD-1 alone, suggesting that SKWX301 likely harbors superior neutralization capacity compared to the reference anti-Tim4 antibody (Fig. 4A). However, although combination treatment with anti-PD-1 plus RMT4-53 did not result in significant changes in tumor volume compared to anti-PD-1 alone, overall survival was largely improved. Interestingly, high doses of SKWX301 (250 μg) did not act synergistically with anti-PD-1. This could be due to technical reasons, such as antibody aggregation, resulting in less molecules being able to actively bind TIM-4 or to biological reasons, such as compensatory T cell mechanisms. The dose sensitivity warrants further investigation, starting with a close examination of the impact of different doses of SKWX301 on tumor infiltrating lymphocytes given the role of TIM-4 in T cell homeostasis. Repetition of the current animal study is necessary to validate the low dose effect of SKWX301. Furthermore, understanding how SKWX301 is functionally modifying the tumor microenvironment at different doses will be critical for further drug development. The current study examined naïve tumors; this is a clear limitation of the study as we would like to see how SKWX301 would function in treatment resistance tumors. The use of a humanized mouse model with patient derived xenografts would allow us to better test SKWX301 feasibility in humans. Specifically, because SKWX301 was developed against human TIM-4 having a system that more accurately mimics the human condition will be necessary to examine the efficacy of SKWX301 in future studies.

To begin to elucidate the mechanism of action by which SKWX301 potentiates anti-tumor immunity, we utilized predictive docking software [36, 37] to investigate how SKWX301 may be interacting with TIM-4. Predictions suggest that TIM-4 may be binding opposite of the integrin binding site and just below the PS binding site (Supplementary Fig. 3). It is possible that SKWX301 binding to TIM-4 may result in TIM-4 conformational changes that function to reduce PS binding. TIM-4 acts as a ligand for both TIM-4 and TIM-1 [12, 38], when TIM-4 binds to TIM-1 it induces TIM-1 phosphorylation activating T cell expansion [38]. One hypothesis is SKWX301 blocks TIM-4 from binding TIM-1 in *in vivo* murine models, leading to decreased T cell expansion and consequently decreased macrophage interactions. Related, SKWX301 may be disrupting hetero- or homo-oligomerization of TIM molecules, which may impact receptor endocytosis. Binding experiments to determine how SKWX301 affects hetero-or homo-oligomerization remains to be explored in the future. Thus, examining T cell expansion and SKWX301 binding dynamics in tumors treated with SKWX301 will be a critical next step to decipher the functional ramifications of the SKWX301-TIM-4 interaction and better understand how SKWX301 acts in the tumor microenvironment to potentiate immunotherapy.

## Conclusions

This study illustrates the potential of phage display and directed protein engineering for rapid biologic drug discovery. Using our rapid antibody discovery platform, we engineered a novel human anti-TIM-4 antibody that effectively inhibits phagocytosis and synergizes with anti-PD-1 inhibitors to block tumor progression in a syngeneic mouse model of colon cancer. Colon cancer represents a disease that has a high frequency of resistance to immunotherapy; however, the need for effective approaches that can improve the efficacy of immunotherapy exists across a number of other oncology indications including NSCLC, melanoma, pancreatic cancer, rectal cancer, and breast cancer among others [16, 18, 39]. In conclusion, these studies demonstrate the specificity of SKWX301 for human and mouse TIM-4 with enhanced biological effect on phagocytosis. SKWX301 represents a promising new potential therapeutic strategy for patients with tumors that exhibit resistance to immunotherapy.

## List of Abbreviations

**Table:** 

T-cell immunoglobulin and mucin domain	TIM
Phosphatidylserine arginine-glycine-aspartic	PS RGD
antigen presenting cells	APCs
non-small cell-lung cancer	NSCLC
danger-associated molecular patterns	DAMPs
cytotoxicity T lymphocyte	CTL
hexahistidine	6X His
peptides with lengths of twenty amino acids	20mer
Complementary-determining regions	CDRS

## Supplementary Material

Slide1_tbae026

Slide2_tbae026

Slide3_tbae026

Slide4_tbae026

supplementary_figure_legends_tbae026

## Data Availability

Materials available from the corresponding author, KF, upon reasonable request.

## Animal research statement

Animal experiments took place at the University of Miami Miller School of Medicine. Animals used in this study adhered to the highest standards of regulatory compliance and ethical guidelines and approved by IACUC (# 21-083). Antibody half-life study was performed at Charles River Laboratories. The study was reported in compliance with ARRIVE guidelines. No patients were included.

## Authors information

Karla Frietze, email: Kfrietze@skwxbio.com

Neil Goldstein, email: ngoldstein@skwxbio.com
