# Supplementary figures and images for: Directed protein engineering identifies a human TIM-4 blocking antibody that enhances anti-tumor response to checkpoint inhibition in murine colon carcinoma
Source: Antib Ther. 2024 Sep 23;7(4):324–34. doi: 10.1093/abt/tbae026 (PMC11638112; doi:10.1093/abt/tbae026)

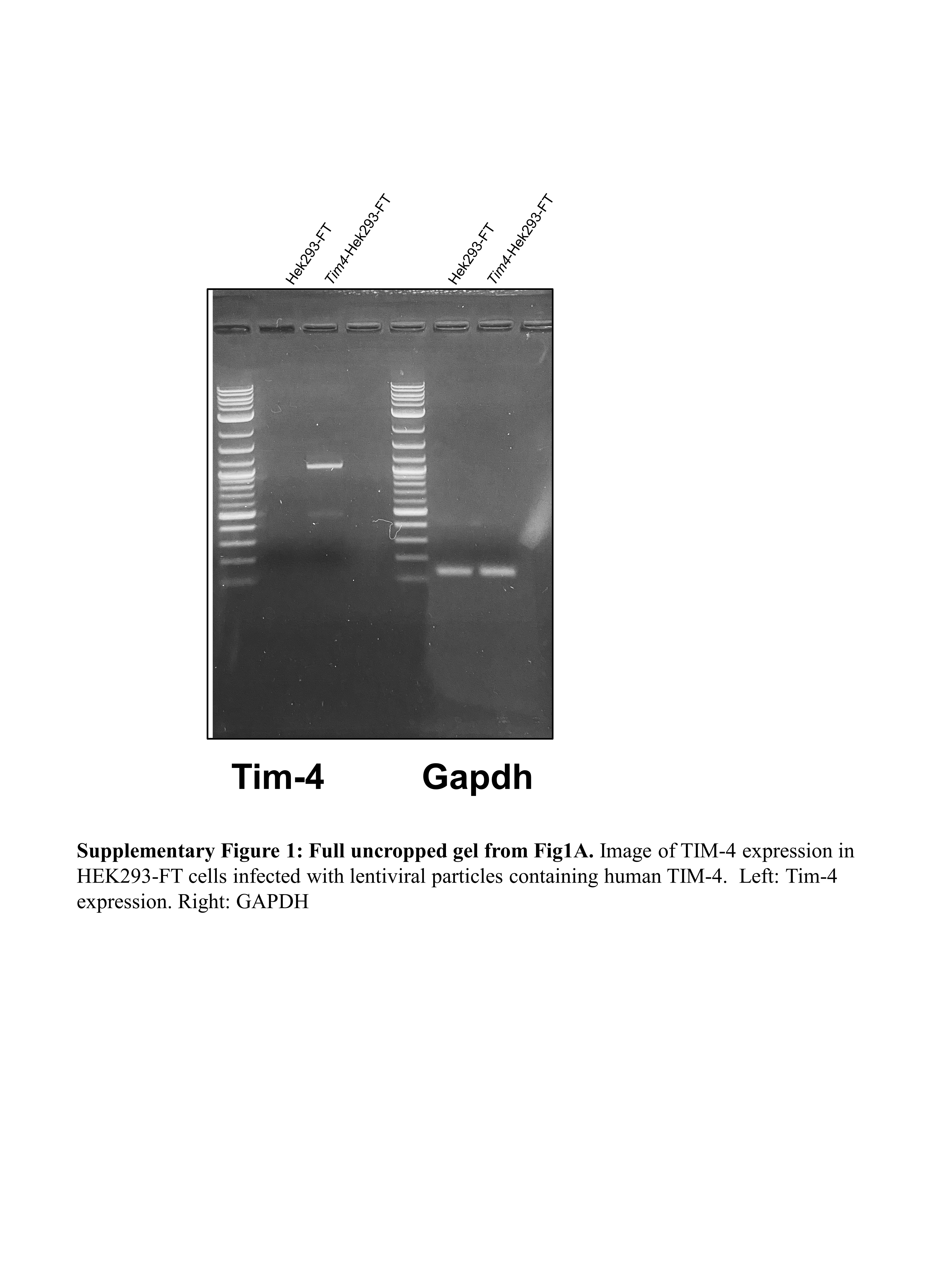

Supplement: Slide1_tbae026 [file slide1_tbae026.jpeg]

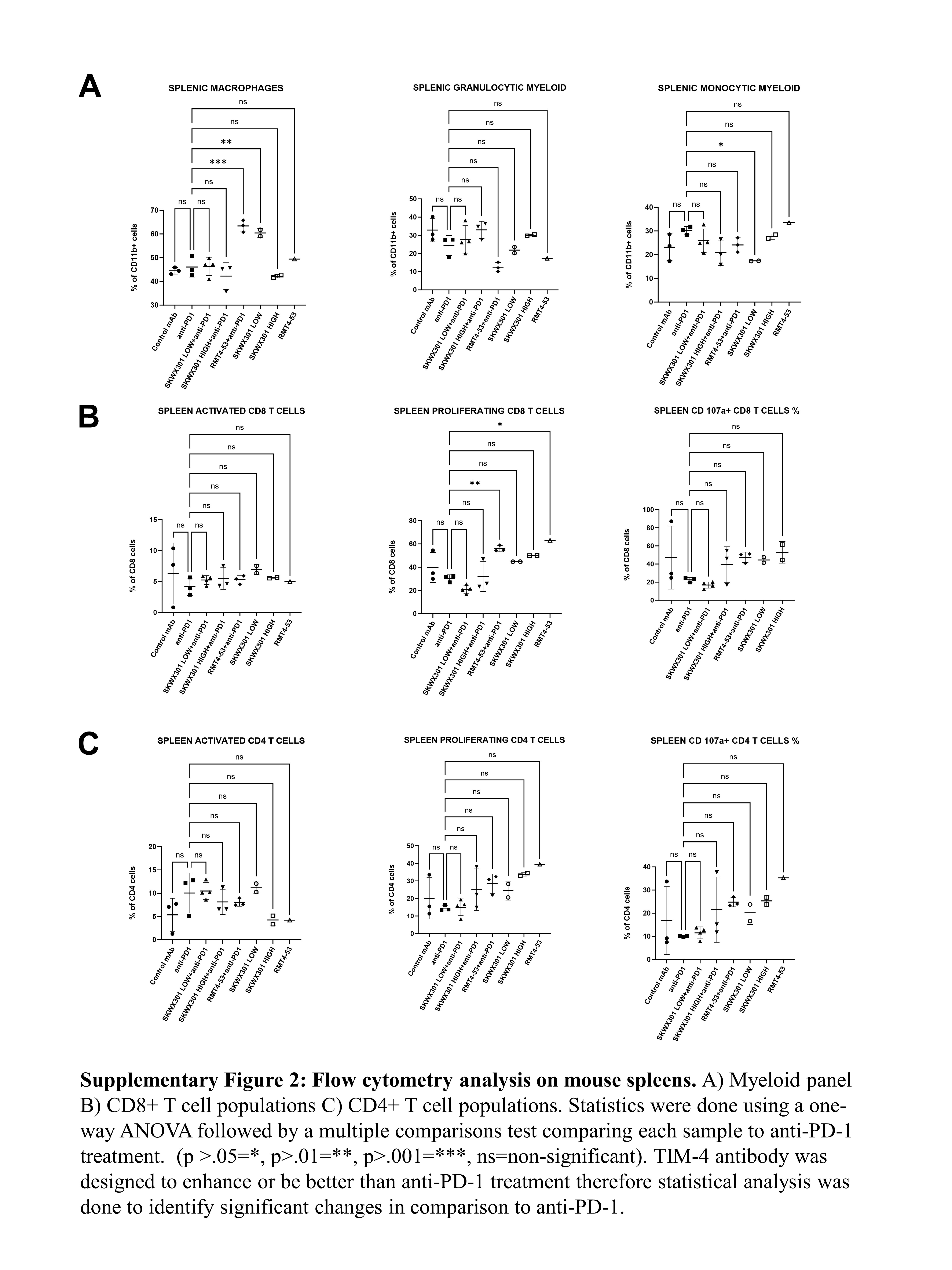

Supplement: Slide2_tbae026 [file slide2_tbae026.jpeg]

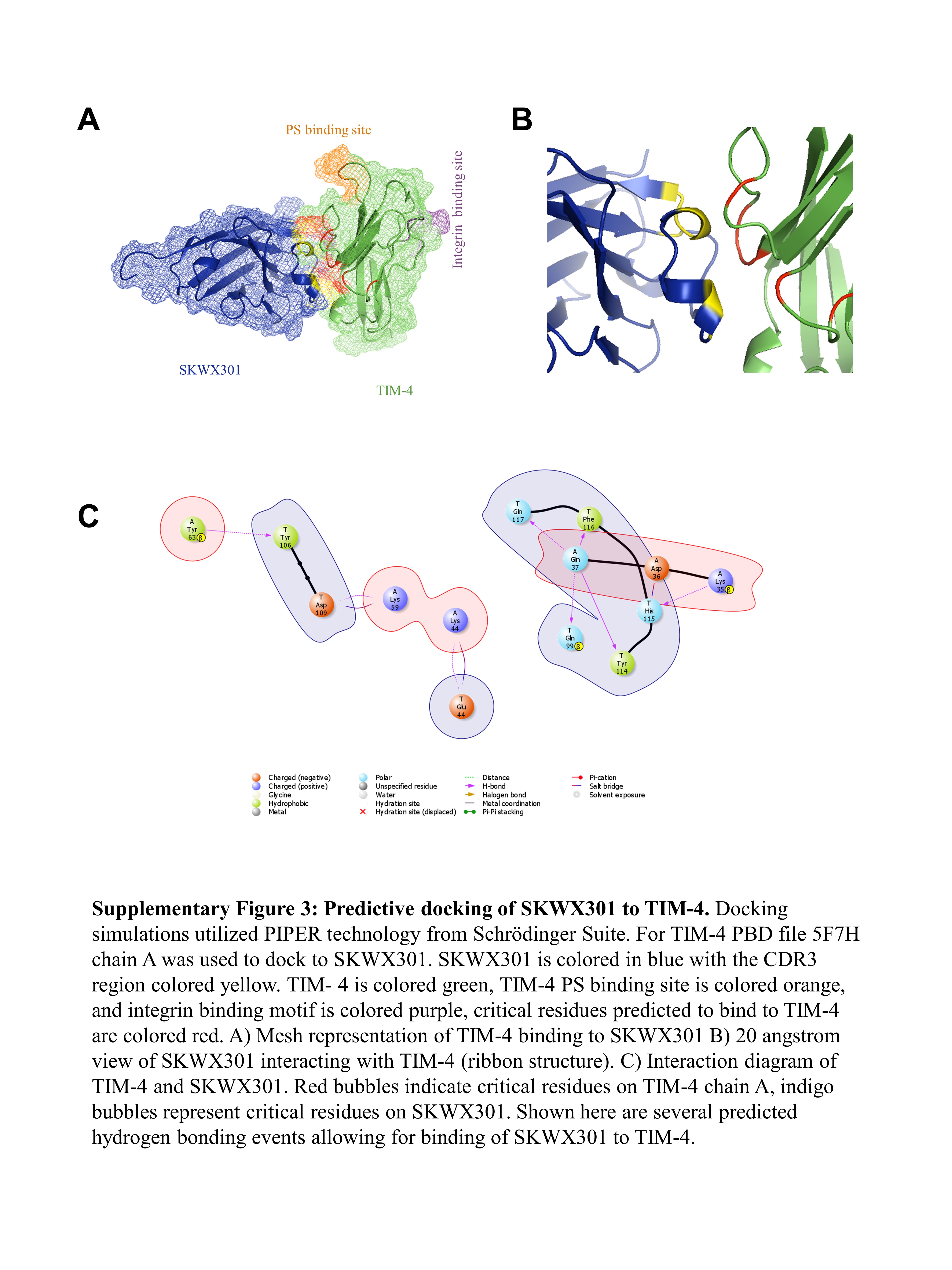

Supplement: Slide3_tbae026 [file slide3_tbae026.jpeg]

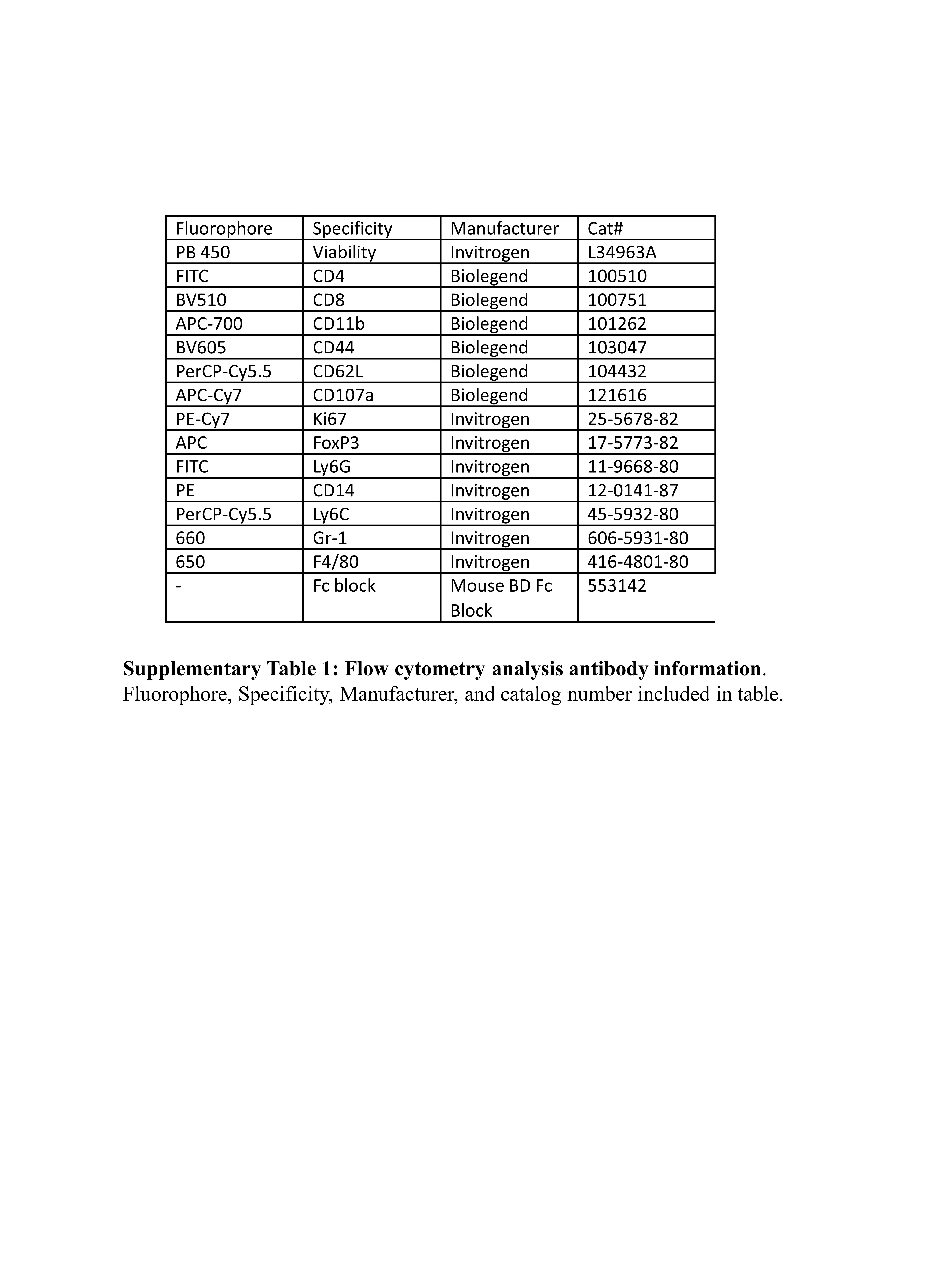

Supplement: Slide4_tbae026 [file slide4_tbae026.jpeg]
