# Supplementary material for: Directed protein engineering identifies a human TIM-4 blocking antibody that enhances anti-tumor response to checkpoint inhibition in murine colon carcinoma
Source: Antib Ther. 2024 Sep 23;7(4):324–34. doi: 10.1093/abt/tbae026 (PMC11638112; doi:10.1093/abt/tbae026)
Supplement: supplementary_figure_legends_tbae026 [file supplementary_figure_legends_tbae026.docx]

**Appendices/Supplemental Material**

**Supplementary Figure 1: Full uncropped gel from Fig1A.** Image of TIM-4 expression in HEK293-FT cells infected with lentiviral particles containing human TIM-4. Left: Tim-4 expression. Right: GAPDH.

**Supplementary Figure 2: Flow cytometry analysis on mouse spleens. A)** Myeloid panel **B)** CD8+ T cell populations **C)** CD4+ T cell populations. Statistics were done using a one-way ANOVA followed by a multiple comparisons test comparing each sample to anti-PD-1 treatment. (p >.05=*, p>.01=**, p>.001=***, ns=non-significant). TIM-4 antibody was designed to enhance or be better than anti-PD-1 treatment therefore statistical analysis was done to identify significant changes in comparison to anti-PD-1.

**Supplementary Figure 3: Predictive docking of SKWX301 to TIM-4.** Docking simulations utilized PIPER technology from Schrödinger Suite. For TIM-4 PBD file 5F7H chain A was used to dock to SKWX301**.** SKWX301 is colored in blue with the CDR3 region colored yellow. TIM-

4 is colored green, TIM-4 PS binding site is colored orange, and integrin binding motif is colored purple, critical residues predicted to bind to TIM-4 are colored red. **A)** Mesh representation of TIM-4 binding to SKWX301 **B)** 20 angstrom view of SKWX301 interacting with TIM-4 (ribbon structure). **C)** Interaction diagram of TIM-4 and SKWX301. Red bubbles indicate critical residues on TIM-4 chain A, indigo bubbles represent critical residues on SKWX301. Shown here are several predicted hydrogen bonding events allowing for binding of SKWX301 to TIM-4.

**Supplementary Table 1: Flow cytometry analysis antibody information.** Fluorophore, Specificity, Manufacturer, and catalog number included in table.
